# Leukemia-Related Mortality in Inner Mongolia, 2008–2012

**DOI:** 10.5539/gjhs.v8n7p146

**Published:** 2015-11-18

**Authors:** Zhihui Hao, Yongsheng Chen, Yongjun Xu, Maolin Du, Ying Wang, Qing Zhang, Heixiao Bai, Juan Sun

**Affiliations:** 1 Department of Medical Records, Inner Mongolia People’s Hospital, Hohhot, China; 2 School of Public Health, Inner Mongolia Medical University, Hohhot, China; 3 International Mongolia Hospital of Inner Mongolia, Hohhot, China; 4 Department of Respiratory, Inner Mongolia Medical University Affiliated Hospital, Hohhot, China

**Keywords:** leukemia, mortality, risk factors

## Abstract

In this study, we aimed to determine the leukemia-related mortality rates and associated sociodemographic characteristics in the Inner Mongolia region of China. We obtained data for the period 2008–2012 from the Death Registry System maintained by the Inner Mongolia Centers for Disease Control and Prevention. We computed the percentages of leukemia-related deaths and controls diagnosed by various methods and at different levels of hospitals. The χ^2^ test was used to examine differences in leukemia-related mortality according to sex. We also calculated potential years of life lost (PYLL) and average years of life lost. Unconditional logistic regression models were used to analyze the effect of sociodemographic characteristics. The sex-adjusted leukemia-related mortality rate was 3.74/100 000. The mortality rate in men (4.27/100 000) was significantly higher than that in women (3.17/100 000), as was the respective PYLL (8040.5 vs. 6000.5 person-years). Mortality increased with increasing age in both men and women. The highest mortality rate was observed in those over 70 years of age for both men (18.36/100 000) and women (7.68/100 000). Men with a higher education level showed an increased risk of leukemia (odds ratio [OR] = 1.45, 95% confidence interval [CI] = 1.02–2.07, P = 0.04). In men, unemployment was associated with leukemia-related death (OR = 0.63, 95% CI = 0.42–0.95, P = 0.03). The leukemia-related mortality rate in Inner Mongolia was higher than that worldwide and that in China. A higher level of education and unemployment were associated with leukemia-related mortality in Inner Mongolia.

## 1. Introduction

Leukemia is the general term for hematological cancers occurring in the tissues responsible for blood formation, and has various clinical and pathological presentations (Bahram et al., 2013; Goldman & Schafer, 2012). Leukemia is an urgent concern in modern society because of its relatively high incidence and its association with poor survival in most cases (Modak et al., 2011). According to the World Health Organization, leukemia is among the 15 most common forms of cancer in adults (Kampen, 2012). Leukemia has been reported or proposed to be associated with occupational status, education level, marital status (Musarezaie, Khaledi, Esfahani, & Ghaleghasemi, 2014), and area of residence (Sinner, Cerhan, Folsom, & Ross, 2005).

The leukemia-related mortality rate has increased greatly in areas with industrial and relatively rapid economic development. A slowly increasing trend in leukemia-related mortality has been observed in Shandong Province, China, during the last 35 years (Li, Yu, & Wang, 2010). Furthermore, an increasing trend in leukemia-related mortality has been reported in an Iranian population, specifically in those of older age and male sex (Fazeli et al., 2013). Leukemia-related deaths have also increased dramatically over the last 40 years in the United States (Blessed, 2011). Thus, it is a major health problem worldwide, and Inner Mongolia is no exception. The aim of this study was to assess the leukemia-related mortality rates and associated sociodemographic characteristics of a large population in Inner Mongolia, China.

## 2. Methods

### 2.1 Data Source

We obtained data for the period 2008–2012 from the Death Registry System (DRS) maintained by the Inner Mongolia Centers for Disease Control and Prevention (CDC). Data from eight monitoring points were used in the current study. Five of these were from the DRS established by the Chinese Ministry of Health, and the remaining three were from the DRS established by the Inner Mongolia CDC (Xin et al., 2014). The eight monitoring points were as follows: Yakeshi City, Kailu County, Bairin Youqi, Sonid Youqi, Muslims District, Tumd Youqi, Ejin Horo Qi, and Linhe District. The population of the eight monitoring points was 12 million. The cause of death was coded according to the International Classification of Disease-10th Revision (ICD-10). The ICD-10 codes for leukemia used in this study were as follows: C91, lymphoid leukemia; C92, myeloid leukemia; C93, monocytic leukemia; C94, other leukemias of a specified cell type; and C95, leukemia of an unspecified cell type. Deaths randomly selected from among those with ICD-10 codes of V01–X59 (accidents), X60–X84 (intentional self-harm), X85–Y09 (assault), and Y10–Y34 (event of undetermined intent) were used as controls. The controls were matched with the leukemia-related deaths for time of death, area of death, sex, and age (±2 years) in a 1:1 ratio.

Marital status, occupational status, education level, and residence (urban/rural) were chosen as indicators to assess the demographic characteristics of leukemia-related mortality (Guo et al., 2015). All hospitals in Inner Mongolia were classified into four levels: provincial, municipal, county, and township. A pathological diagnosis could be provided by all four hospital levels. Diagnostic methods in this study included pathological, clinical, surgical, and postmortem diagnoses. Clinical diagnoses included imaging, pathological anatomy, and pathophysiology diagnoses. Surgical and postmortem diagnoses were also employed (Hu et al., 2014).

### 2.2 Statistical Analysis

We computed the percentages of leukemia-related deaths and controls according to various methods and at different levels of hospitals in each of the monitoring points during the 5-year study period. Leukemia-related mortality (per 100 000) was calculated according to sex. The χ^2^ test was used to examine differences in leukemia-related mortality between sexes. We computed potential years of life lost (PYLL) and average years of life lost (AYLL) according to sex. PYLL is used to highlight premature mortality by estimating the average time a person would have lived had he or she not died prematurely. AYLL is the average of the difference between the expected age and the actual age at death due to cancer. Unconditional logistic regression models were used to analyze the effect of sociodemographic characteristics. Odds ratios (ORs) and corresponding 95% confidence intervals (CIs) were computed. Leukemia-related deaths were matched with controls for age and sex in the logistic regression analysis. Marital status was classified as married or unmarried (as reference). Occupational status was classified as employed or unemployed (as reference). The level of education was classified as middle school and higher education or illiterate and primary school education (as reference). Residence was classified as urban or rural (as reference). An OR of >1 was considered to indicate an increased risk, and an OR of <1 was considered to indicate a decreased risk. All statistical analyses were performed using Microsoft Excel and SPSS version 13.0 statistical software, and the level of significance was set at P < 0.05.

## 3. Results

Table 1 shows the percentages of leukemia-related deaths and controls diagnosed by different diagnostic methods and at different levels of hospitals. More than 80% of leukemia cases were diagnosed in provincial or municipal hospitals. Approximately 99% of leukemia cases and more than 95% of control cases were diagnosed pathologically.

**Table 1:** Percentages of Leukemia-related Deaths in Inner Mongolia during 2008–2012

	Leukemia-related deaths n(%)	Controls n(%)
**Diagnostic method**		
Clinical and laboratory examinations	259(57.7)	167(37.2)
Clinical	124(27.6)	193(43)
Pathology	57(12.7)	17(3.8)
Surgery	2(0.4)	5(1.1)
Postmortem examination	5(1.1)	61(13.6)
Unknown	2(0.4)	6(1.3)
Total	449(100)	449(100)
**Highest diagnostic institution**		
Provincial hospital	160(35.6)	52(11.6)
Municipal hospital	220(49)	120(26.7)
County-level hospital	62(13.8)	215(47.9)
Township-level hospital	7(1.6)	62(13.8)
Total	449(100)	449(100)

**Figure 1:**
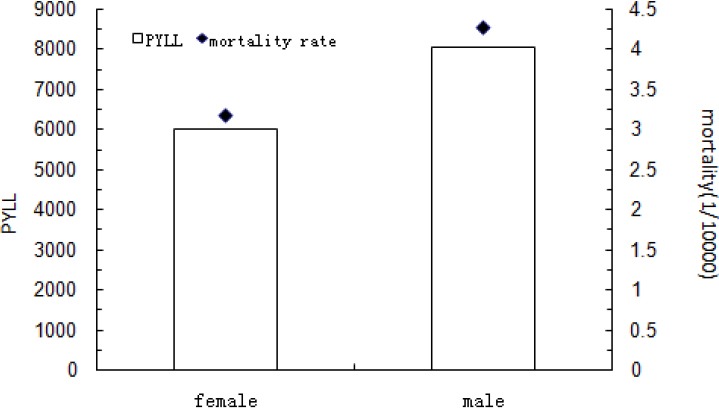
Mortality and PYLL for Leukemia by Sex in Inner Mongolia during 2008–2012 PYLL = potential years of life lost

In total, 449 leukemia-related deaths (264 men and 185 women) were recorded at the eight monitoring points of Inner Mongolia during 2008–2012. The sex-adjusted leukemia-related mortality rate was 3.74/100 000. The PYLL was 14 041 person-years, and the AYLL was 31.27 person-years. Figure 1 shows the mortality and PYLL for leukemia by sex. The mortality rate in men (4.27/100 000) was significantly higher than that in women (3.17/100 000; χ^2^ = 9.628, P < 0.05), with a male-to-female ratio of 1.4. The PYLL was also higher in men than in women: 8040.5 and 6000.5 person-years for men and women, respectively.

**Figure 2:**
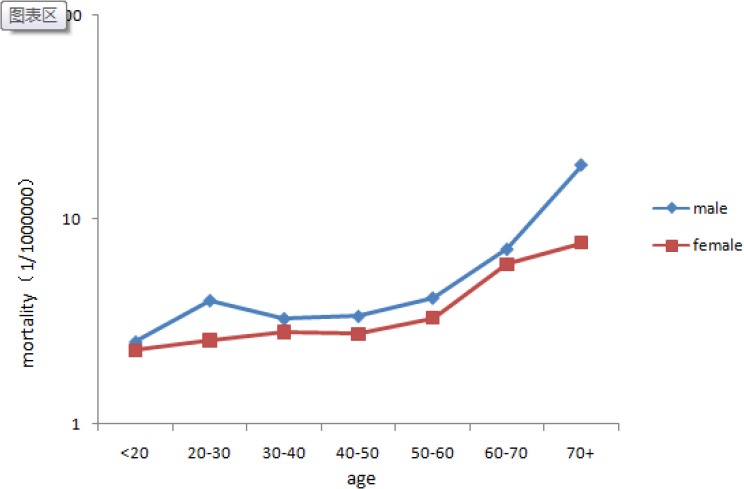
Age-related Mortality Rates for Leukemia by Sex in Inner Mongolia during 2008–2012

The number of leukemia-related deaths in those over 70 years was 52 among men and 22 among women. The age-related mortality rates for leukemia by sex are presented in a semilogarithmic chart in Figure 2. Mortality increased with increasing age in both men and women. Mortality was the highest in those over 70 years of age for both men (18.36/100 000) and women (7.68/100 000), with the rate in men being more than two times higher than that in women.

Table 2 shows the results of multivariate analyses of sociodemographic characteristics and the risk of leukemia in men and women separately. Employment was associated with leukemia-related death in men (OR = 0.63, 95% CI = 0.42–0.95, P = 0.03). Men with a higher education level had an increased risk of leukemia (OR = 1.45, 95% CI = 1.02–2.07, P = 0.04). There were no statistically significant relationships between the factors we studied and leukemia-related death in women (P > 0.05).

**Table 2:** Logistic Regression Analysis of Sociodemographic Characteristics for Leukemia Mortality by Sex

	Women				Men			

Characteristic	n(%)	OR	95% CI	P	n(%)	OR	95% CI	P
**Marital status**				0.33				0.86
Unmarried	66(35.7)	1			89(33.7)	1		
Married	119(64.3)	1.27	0.76–2.04		175(66.2)	1.03	0.71–1.52	
**Occupation**				0.29				0.03
Unemployed	64(34.6)	1			87(33.0)	1		
Employed	121(65.4)	0.76	0.46–1.26		177(67.0)	0.63	0.42–0.95	
**Education level**				0.45				0.04
Illiterate and primary school	84(45.4)	1			96(36.4)	1		
Middle school and higher	101(54.6)	1.18	0.77–1.79		168(63.6)	1.45	1.02–2.07	
**Area of residence**				0.84				0.24
Rural	37(46.8)	1			51(19.3)	1		
Urban	148(50.9)	1.06	0.63–1.78		213(80.7)	0.76	0.48–1.2	

*Note*. OR = odds ratio; CI = confidence interval.

## 4. Discussion

The sex-adjusted leukemia-related mortality rate in Inner Mongolia was 3.74/100 000 in 2008–2012. This rate is higher than the worldwide rate (3.4/100 000) (Ferlay et al., 2010) and the rate in China (3.27/100 000) (Chen et al., 2014). The leukemia-related mortality rate of men in Inner Mongolia was lower than that of men in developed areas of the world (4.8/100 000) but higher than that of men in developing areas of the world (3.7/100 000). However, the leukemia-related mortality rate of women in Inner Mongolia was similar to that of women in developed and developing areas of the world (both 2.9/100,000) (Ferlay et al., 2010). The mortality rate in men was significantly higher than that in women in our study, which is in agreement with findings of previous studies (Liu, Zhao, & Chen, 2013). Correspondingly, the PYLL was higher in men than in women.

Leukemia-related death was observed in all age groups. Mortality was the highest in an older age group (>70 years) in both men and women. This result is consistent with that of a study in the United States that reported a leukemia-related mortality rate of 2.9/100 000 versus 60/100 000 in men aged <65 versus ≥65 years, respectively, and 2.0/100 000 versus 32/100 000 in women aged <65 versus ≥65 years, respectively (Igissinov et al., 2014). Compared with the mortality rate, which is usually higher in the elderly, the AYLL lends greater weight to age for diseases that result in younger deaths and less weight to age for diseases affecting the elderly (Pham, Fujino, Matsuda, & Yoshimura, 2010). In our study, the AYLL was 31.27 person-years, which was higher than that reported in a previous study from China, in which the AYLL for leukemia was the highest, even though leukemia-related mortality was lower than mortality associated with other cancers (Wang & Xiaoshu, 2010). These results highlight the seriousness of premature death due to leukemia.

Men with a higher level of education had an increased risk of leukemia. Several studies have suggested a relationship between educational level as a socioeconomic status indicator and cancer mortality (Albano et al., 2007). Unemployment was associated with an increase in the risk of leukemia-related death in men. Unemployment has been associated with greater mental strain and a higher standardized mortality (Jin, Shah, & Svoboda, 1995), and has been shown to be associated with an increased likelihood of colorectal cancer death (Xin et al., 2014). Although unemployment is considered an important issue, the possible adverse impact on physical and mental health has not yet been elucidated (Linn, Sandifer, & Stein, 1985).

Our study has a limitation. Because the data we obtained did not include any etiological factors related to leukemia, we could not study the deep-rooted causes associated with mortality in leukemia patients.

## 5. Conclusion

In this study, we determined the characteristics of leukemia in Inner Mongolia during the period 2008–2012. Both the mortality rate and PYLL were significantly higher in men than in women during this period. The risk of leukemia increased with an increase in age in both men and women, with mortality being the highest in those over 70 years of age in both sexes. In men, both a higher education level and unemployment were associated with an increased risk of leukemia. Further studies are needed to assess the more recent trends in leukemia-related mortality.
